# Comparative analysis of codon usage bias revealed natural selection-driven evolution and host adaptation of infectious haematopoietic necrosis virus

**DOI:** 10.1093/ve/veag062

**Published:** 2026-09-21

**Authors:** Yu-Meng Yang, Fang Liu, Liu-Yi Yu, He Sun, Adili Abudu, Jian-Yong Hu, Yue Zhuo, Cui-Lan Hao, Cheng Yue

**Affiliations:** College of Veterinary Medicine, Xinjiang Agricultural University, 311 Nongda East Road, Saybag District, Urumqi 830052, Xinjiang, China; College of Veterinary Medicine, Xinjiang Agricultural University, 311 Nongda East Road, Saybag District, Urumqi 830052, Xinjiang, China; College of Veterinary Medicine, Xinjiang Agricultural University, 311 Nongda East Road, Saybag District, Urumqi 830052, Xinjiang, China; College of Veterinary Medicine, Xinjiang Agricultural University, 311 Nongda East Road, Saybag District, Urumqi 830052, Xinjiang, China; Xinjiang Uygur Autonomous Region Fisheries Development Center (Xinjiang Uygur Autonomous Region Fisheries Science Research Institute), 614 Xihong West Road, Saybag District, Urumqi 830099, Xinjiang, China; Xinjiang Uygur Autonomous Region Fisheries Development Center (Xinjiang Uygur Autonomous Region Fisheries Science Research Institute), 614 Xihong West Road, Saybag District, Urumqi 830099, Xinjiang, China; College of Veterinary Medicine, Xinjiang Agricultural University, 311 Nongda East Road, Saybag District, Urumqi 830052, Xinjiang, China; College of Veterinary Medicine, Xinjiang Agricultural University, 311 Nongda East Road, Saybag District, Urumqi 830052, Xinjiang, China; Xinjiang Regional Key Laboratory of Clinical Veterinary Medicine Research, 311 Nongda East Road, Saybag District, Urumqi 830052, Xinjiang, China; Xinjiang Key Laboratory of New Drug Research and Development for Herbivorous, 311 Nongda East Road, Saybag District, Urumqi 830052, Xinjiang, China; College of Veterinary Medicine, Xinjiang Agricultural University, 311 Nongda East Road, Saybag District, Urumqi 830052, Xinjiang, China

**Keywords:** infectious haematopoietic necrosis virus, codon usage bias, phylogeny, host adaptation, population dynamics

## Abstract

The infectious haematopoietic necrosis virus (IHNV) is an important threat for global salmonid aquaculture, particularly *Oncorhynchus mykiss*. Understanding molecular mechanisms of evolution and host adaptation is crucial. This study comparatively analyses codon usage bias (CUB) between IHNV genotypes and their relations to host adaptation and evolutionary forces. Phylogenetic analysis of IHNV reveals topological differences based on whole-genome and G gene. Temporal analysis mapped major genotypic diversification to the 1950s–1970s. At the whole-genome and G gene levels, the most frequently preferred codons were related to the encoding of Leu (CTC), Ser (TCC), Pro (CCC), and Thr (ACC) across all genotypes (RSCU > 1.6). All data points fall below the expected effective number of codons (ENC) curve in ENC-plot. Codon usage for every genotype departs from the PR2-plot origin (0.5, 0.5) to varying extents in both the G gene and the whole genome. For whole-genome datasets, the selection contribution metric values for genotypes U, J, and E were 45.22%, 89.23%, and 99.52%, respectively, in Neutrality plots. Correspondingly, the selection contribution metric for G gene genotypes L, J, U, M, and E were 56.49%, 68.49%, 93.57%, 96.45%, and 85.52%, respectively. The CpG, GpC, GpT, TpT, and TpA were markedly underrepresented (Pxy < 0.78), while CpA, GpA, and TpC were overrepresented in both the G gene and the whole genome (Pxy > 1.25). Codon adaptation index analysis shows a conserved optimal adaptation of all IHNV genotypes to salmonid hosts (rainbow trout and Chinook salmon). Bayesian skyline reconstruction shows genotype-specific population dynamics with expansion phases associated to intensive aquaculture. These results provide a molecular framework for understanding IHNV epidemiology and host specificity.

## Introduction

Infectious haematopoietic necrosis virus (IHNV), belonging to the genus *Novirhabdovirus* within the family Rhabdoviridae, is the causative agent of a severe and often lethal disease in salmonid fishes, particularly *Oncorhynchus mykiss* (Kurath et al. 2003). Infectious haematopoietic necrosis is characterized by high mortality rates in juvenile fishes leading to economic losses for the global trout aquaculture industry (Furey et al. 2021). The virus–host interaction between IHNV and *O. mykiss* is a dynamic interface that determines the infection outcome (Penaranda et al. 2009), which can range from acute mortality to persistent or subclinical infection. Understanding the molecular mechanisms of IHNV virulence and host adaptation is therefore essential for effective control. In recent years, the analysis of viral codon usage bias (CUB) has become an efficient tool for investigating host adaptation, evolutionary constraints, and possible attenuation (Ventoso 2025). Our study aim to comparatively analyse CUB of IHNV and its primary host in order to understand the viral adaptation patterns, the implications for viral evolution, and pathogenesis.

All organisms exhibit CUB, a non-random usage of synonymous codons (Parvathy et al. 2022). This bias arises from the interplay of mutation pressure with natural selection (Bulmer 1991). Mutation pressure refers to random nucleotide substitutions during genome replication, and natural selection actively favours codons that enhance translational efficiency or host adaptation. For RNA viruses such as IHNV with compact genomes and high mutation rates, CUB is crucial in translation efficiency, replication speed, and fitness. When a virus infects a host, the CUB of the virus and the host can limit translation efficiency, blocking cross-species transmission and efficient replication (Ventoso 2025). Conversely, convergence or adaptation of viral codon usage towards the host pattern is usually seen as an evolution scheme to hijack the host translation machinery effectively (Tenbusch et al. 2010, Zhi et al. 2010, Albers and Czech 2016). IHNV has known genetic diversity and exists in different genotypes with different host specificities and virulence profiles (Biacchesi 2011, Kell et al. 2013).

Recent studies have focused on IHNV genome organization, phylogenetic classification into major U, M, L, E, J genotypes and functions of its six genes (N, P, M, G, NV, L) (Kurath et al. 1985, Morzunov et al. 1995). Epidemiological studies have mapped global distribution of the genotypes, and link genotype M to most epidemics in *O. mykiss*, genetic markers for viral transmission (G gene) (Garver et al. 2006). The G gene of IHNV encodes the viral glycoprotein, which is the major surface antigen responsible for host cell receptor binding and membrane fusion, thereby playing a key role in viral infectivity and host immune recognition (Huang et al. 1996). Consequently, the G gene of IHNV is a primary target for neutralizing antibody responses and is frequently analysed in molecular epidemiological studies as a key genetic marker for viral evolution and adaptation (Engelking and Leong 1989, Huang et al. 1996). Virological codon usage analysis has also developed. Studies on human and animal viruses (influenza, Zika, coronavirus) have shown strong correlations between codon adaptation to host and viral pathogenicity, tissue tropism, and evolutionary trajectory (Butt et al. 2016, Nambou and Anakpa 2020, Nambou et al. 2022).

However, many problems and technical problems remain for the IHNV-*O. mykiss* system. First, there is no systematic genome-wide analysis of codon usage patterns for IHNV. Many studies focus on single genes (e.g. G gene) and therefore not on the collective and gene-specific CUB of the entire genome. Second, it is unclear how codon usage patterns differ between different IHNV genotypes and whether these differences are related to their host range and virulence. Is the success of genotype M in rainbow trout reflected in a codon usage profile more optimized for this host than genotype U? Third, the relative roles of natural selection and mutation pressure in framing the CUB of IHNV remain to be studied. As an RNA virus with a A/U rich genome, composition constraint (mutation pressure) is likely strong, but how natural selection has played a role in tailoring codon usage for host adaptation needs quantitative dissection.

The innovation and purpose of this work is to directly address these gaps. The main innovation is to conduct the first comprehensive comparative codon usage analysis, where all of the coding sequences of different IHNV isolates (with major genotypes) are directly comparably compared to genomic and predicted translational landscape of three different hosts: *O. mykiss, Oncorhynchus tshawytscha,* and *Esox lucius.* We move beyond single-gene analysis to provide a complete view of viral CUB. Methodologically, we employed a series of robust analytical tools, including relative synonymous codon usage (RSCU), effective number of codons (ENC), codon adaptation index (CAI), relative codon deoptimization index (RCDI), similarity index (SiD), and parity rule 2 (PR2), to dissect the dynamics of CUB and to assess viral adaptability.

The purpose of this work is three-fold: (i) characterize and compare codon usage patterns across the genome of IHNV and between different IHNV genotypes and identify genes or regions with strongest or most atypical bias; (ii) quantify the extent to which IHNV adapts to fish hosts by CUB and determine whether CUB is mutational pressure or natural selection driving its adaptation behaviour; (iii) correlate codon usage patterns with known biological features of IHNV strains (genotype, host of origin, reported virulence) to provide testable hypotheses on CUB in host adaptability and pathogenicity. Finally, this study will offer new insights into molecular evolution of IHNV, reveal genetic limitations on its trans-species transmission, and identify candidate genomic features that may influence its virulence. This knowledge could inform future strategies for monitoring emerging strains, and even rational design of live-attenuated vaccines by deliberate de-optimization, as successfully done in other viral systems (Cai et al. 2020). Finally, this work will further deepen our understanding of the fundamental mechanisms governing host-pathogen co-evolution in aquatic environments.

## Materials and methods

### Sequence collection and processing

The complete genomic and G gene sequences of IHNV were acquired from NCBI (https://www.ncbi.nlm.nih.gov/genbank/). Prior to all downstream analyses, strict quality control was implemented to ensure sequence reliability. Sequences were excluded if they failed to meet the predefined length and base-quality thresholds: complete genome sequences shorter than 10 000 bp or G gene coding sequences shorter than 1300 bp were discarded, as were sequences with an overall proportion of ambiguous N bases exceeding 5% or containing more than 10 consecutive N residues. The remaining sequences were further manually curated to correct mislabelling, resolve taxonomic inconsistencies, and remove potentially contaminated sequences. After quality control filtering, a total of 41 IHNV complete genome sequences and 67 IHNV G gene sequences were retained for subsequent analyses. The complete genome dataset comprised 2 sequences of genotype M, 10 of genotype JRT, 4 of genotype U, and 25 of genotype E, whereas the G gene dataset consisted of 6 sequences of genotype L, 10 of genotype U, 7 of genotype M, 16 of genotype JRT, and 28 of genotype E. The accession numbers and basic information of all included whole-genome sequences are listed in Supplementary Table S1, and those of the G-gene sequences are listed in Supplementary Table S2.

### Phylogenetic and temporal evolutionary trajectory of IHNV

Forty-one complete genome sequences of IHNV and 67 G gene nucleotide sequences of IHNV representing the 5 known genogroups (U, M, L, E, and J) of IHNV were selected to construct phylogenetic trees separately. As the NCBI has no complete genome data for genogroup L of IHNV, the genogroup U of IHNV was used as the outgroup for the genome-based tree, whereas genogroup L was used as the outgroup for the G-gene-based tree. Multiple sequence alignments of IHNV were performed by the MAFFT plugin implemented in PhyloSuite 1.2.3 (Katoh et al. 2002). After alignment, the resulting sequences were automatically trimmed with the trimAl plugin to remove ambiguously aligned regions and gaps (Capella-Gutiérrez et al. 2009). Based on the trimmed alignment files, maximum likelihood (ML) phylogenetic trees were reconstructed by IQ-TREE 1.6.8 (Nguyen et al. 2015). The best-fit nucleotide substitution models of ML were automatically evaluated and chosen on ModelFinder with the Bayesian information criterion (BIC) (Kalyaanamoorthy et al. 2017). Branch support was evaluated by the ultrafast bootstrap method with 5000 replicates along with the SH-aLRT branch test with 1000 replicates (Zhang et al. 2019). The final ML trees were visualized and displayed with the iTOL web server (https://itol.embl.de/).

Homologous recombination screening was conducted on the trimmed alignments using RDP4 v4.101 to rule out recombination as a confounding factor for phylogenetic inference and codon usage evolution analysis. Seven independent algorithms (RDP, GENECONV, BootScan, MaxChi, Chimaera, SiScan, and 3Seq) were applied for detection (Martin et al. 2015). Separate analyses were performed for the whole-genome alignment and the isolated G-gene coding sequence alignment. A recombination event was defined as robust if supported by all seven algorithms (Kim et al. 2025).

The temporal evolutionary trajectory of IHNV was analysed based on the G gene sequences, which were aligned and trimmed as described previously. The aligned sequence was imported into BEAUti, the graphical front-end of BEAST 2.7.7 (Drummond and Rambaut 2007). In the ‘Tips’ panel, precise sampling dates from the metadata were assigned to each sequence to calibrate the evolutionary timescale. The core model was configured as follows: the HKY (Hasegawa–Kishino–Yano) model was used for nucleotide substitution (Bouckaert and Drummond 2017). To select the optimal molecular clock model, the coefficient of rate variation (ucldStdev) was adopted as the statistical criterion to compare the strict clock and uncorrelated log-normal relaxed clock (UCLD) models. The 95% highest posterior density (HPD) interval of ucldStdev in the UCLD model did not contain zero, indicating significant substitution rate heterogeneity across lineages and rejecting the constant-rate assumption of the strict clock (Marino et al. 2011). The UCLD relaxed clock model was therefore adopted for subsequent dating analysis. Coalescent Bayesian Skyline was chosen as the tree prior. Two independent Markov Chain Monte Carlo (MCMC) runs were performed in parallel. The MCMC parameters were set with a chain length of 50 000 000 generations, which were sampled every 10 000 iterations (Shi et al. 2025), while other parameters retained their default values in BEAUti. Subsequently, the final executable XML configuration file was generated. This XML file was then executed using BEAST 2.7.7 in the CPU mode. The convergence of the MCMC runs was tested with Tracer 1.7.2. Results were considered reliable and retained for downstream analysis only when the effective sample sizes (ESS) for all key parameters exceeded 200 (Rambaut et al. 2018). The posterior tree samples (.trees files) generated by BEAST were summarized using TreeAnnotator 2.7.7, with the maximum clade credibility tree chosen as the target tree type, to produce a time-calibrated phylogenetic tree annotated with divergence time estimates (Mbangiwa et al. 2018). The final tree was visualized on FigTree 1.4.5 (Chen et al. 2012).

### Codon usage bias analysis

The codon usage composition of the whole genome and G gene of IHNV were determined by CAIcal SERVER (https://ppuigbo.me/programs/CAIcal/) (Puigbò et al. 2008) and CodonW 1.4.2 (Parvathy et al. 2022). The analysis included (i) overall proportions of adenine (A), guanine (G), cytosine (C), and thymine (T); (ii) their frequencies at the third codon position (A3, G3, C3, T3); (iii) AT and GC contents (AT1, AT2, AT3, GC1, GC2, GC3); (iv) overall GC content at synonymous third codon positions (GC3s); (v) ENC; (vi) RSCU. RSCU > 1.6 indicates a preference for codon, while RSCU < 0.6 indicates underrepresentation (Khandia et al. 2024).

The dinucleotide abundance was calculated by EMBOSS (http://www.bioinformatics.nl/emboss-explorer/) as follows:


$$ {\rho}_{XY}=\frac{f_{XY}}{f_X{f}_Y} $$

where *f_XY_*, *f_X_*, and *f_Y_* are the detected frequencies of dinucleotide XY, nucleotides X and Y, respectively. Dinucleotides with relative abundance larger than 1.25 are defined as overrepresented, and those with a value less than 0.78 are classified as underrepresented (Kunec and Osterrieder 2016, Khandia et al. 2019). To exclude confounding effects from codon reading frame constraints, a Monte Carlo resampling analysis was conducted as described by Gu et al. (Gu et al. 2019). For each sequence, synonymous codons were randomly shuffled 1000 times with preserving the original amino acid sequence and the total count of each synonymous codon, and dinucleotides were counted only at codon-codon junctions (position 3-1). The corrected index *ρ*^MC^ was calculated as:


$$ \rho \frac{MC}{xy}=\frac{count\left({xy}_1\right)}{mean\left(\left({x}_i{y}_i\right) random\right)} $$

$$ x, y\epsilon \left\{A,T,C,G\right\} $$

where the numerator is the observed count in the real sequence, and the denominator is the mean count calculated from 1000 random permutations of the same original sequence. A *ρ*^MC^ value significantly less than 1 indicates that the observed dinucleotide depletion is driven by selective pressure rather than inherent coding frame constraints.

### Evolutionary forces shaping CUB of IHNV

Based on nucleotide composition data at the third codon positions, compositional asymmetry indices were computed as:


$$ {X}_{axis}=\frac{G_3}{G_3+{C}_3} $$

$$ {Y}_{axis}=\frac{A_3}{A_3+{T}_3} $$

where *A*_3_, *T*_3_, *C*_3_, and *G*_3_ represent the frequency of Adenine, Thymine, Guanine, and Cytosine at the respective sites. The scatter plot was generated with GraphPad Prism 9.0 (Liu et al. 2020). During neutral evolution, codon usage is expected to be uniform around the central coordinates (0.5, 0.5). Deviation from the central coordinates indicates that selection pressure or mutational bias has an asymmetric effect on the composition (Sueoka 1995).

The ENC plot was computed by plotting the calculated ENC against the GC3s. The expected ENC curve was used to describe the expected curve under neutral mutation pressure. If codon usages were entirely random, the data points would be aligned with the expected curve, which suggests mutational pressure as the main evolutionary force. The data points below the expected curve suggest more natural selection on codon usage (Deb et al. 2020, Rahman et al. 2025). The ENC plot was constructed using the Bioinformatics Cloud platform (http://112.86.217.82:9929/) provided by Nanjing Genepioneer Biotechnology Co. The formula for the expected curve is as follows:


$$ {\mathrm{ENC}}^{\mathrm{expected}}=2+\mathrm{X}+\frac{29}{{\mathrm{X}}^2+{\left(1-\mathrm{X}\right)}^2} $$

where X is defined as the frequency of GC3s.

The neutrality plot was generated with GC3s as the x-coordinate and GC12s as the y-coordinate plotted linearly. Results show that when the slope approaches 1, mutation pressure is supposed to involve in CUB, while a slope near 0 implies more impact from natural selection. When GC12 is not significantly associated with GC3 (*P* > .05), the base compositions of positions 1, 2, and 3 vary independently, probably due to different evolutionary pressures (e.g*.* complex balancing selection or species-specific genomic structure) (Sueoka 1988, Sueoka and Kawanishi 2000). Based on the slope of regression, the relative contribution of natural selection can be estimated using (1-slope) × 100%, which quantifies the relative strength of selection and mutation in codon usage patterns (He et al. 2017).

### Host adaptation of IHNV

CAI, RCDI, and SiD were analysed. CAI is a measure of synonymous CUB that assesses the relative fitness of each codon in a gene (Sharp and Li 1987). RCDI quantifies the extent to which the codon usage of a gene deviates from that of its host organism (Mueller et al. 2006). The host codon usage tables for *O. mykiss*, *O. tshawytscha*, and *E. lucius* were acquired from the codon usage database (https://www.kazusa.or.jp/codon/) (Nakamura et al. 2000), the host RSCU values are calculated as follows:


$$ {RSCU}_i=\frac{n_i}{\frac{1}{k}{\sum}_{j=1}^k{n}_j} $$

where *n_i_* denotes the observed usage count (or frequency value) of the *i*-th codon, *k* represents the total number of synonymous codons for the corresponding amino acid, and ${\sum}_{\mathit{\mathsf{j}}=\mathsf{1}}^{\mathit{\mathsf{k}}}{\mathit{\mathsf{n}}}_{\mathit{\mathsf{j}}}$ indicates the sum of usage counts (or frequencies) across all synonymous codons of the amino acid (Posani et al. 2022). The CAI and RCDI were calculated with CAIcal SERVER (https://ppuigbo.me/programs/CAIcal/). CAI ranges from 0 to 1, and a higher CAI indicates that the target gene predominantly uses the most frequent codons in the host reference set (Sharp and Li 1987). RCDI approaching 1 indicates greater similarity between the host and viral codon usage patterns (Mueller et al. 2006, He et al. 2019).

The SiD/D(A, B) is calculated as follows (Jenkins and Holmes 2003):


$$ R\left(A,B\right)=\frac{\sum_{i=1}^{59}{a}_i{b}_i}{\sqrt{\sum_{i=1}^{59}{\mathrm{a}}_{\mathrm{i}}^2{\sum}_{i=1}^{59}{b}_i^2}} $$

$$ SiD/D\left(A,B\right)=\frac{1-R\left(A,B\right)}{2} $$

where *a*_i and *b*_i denote the RSCUs for the identical codon in the virus and host, respectively. The SiD/D (A, B) varies from 0 to 1, and a lower value suggests a higher degree of similarity in codon usage patterns between the virus and host (He et al. 2019, Shi et al. 2025).

### Genotype clustering and discriminant analysis based on codon usage patterns

An RSCU matrix was constructed based on all viral strains. Principal component analysis (PCA) was then conducted using the functionality within GraphPad Prism 10.4.1 for dimensionality reduction. The first two principal components (PC1 and PC2), explaining the greatest proportion of variance, were extracted. A 2D scatter plot was generated to visually represent the distribution of viral strains in the PC1-PC2 space, with data points coloured according to their respective genotypes (Zang et al. 2017).

### Population dynamics analysis

The population history of IHNV overall and its five genotypes was assessed based on the G gene using Bayesian skyline reconstruction. Prior to the analysis, all datasets were evaluated for temporal signal using TempEst 1.5.3 (Rambaut et al. 2016). Bayesian inference was then performed within BEAST2 2.7.7 following the same operational procedures described in Section 2.2. Upon completion of the runs, convergence of the Markov chains was assessed and Bayesian skyline plots were drawn using Tracer 1.7.2.

### Association analysis between codon usage bias and population dynamics

To verify the association between CUB and viral population expansion/contraction, we conducted stratified analyses across five genotypes to eliminate confounding effects from distinct evolutionary lineages. For each genotype, annual mean values of GC3s and ENC were calculated by sampling year. These annual CUB metrics were matched with the posterior median of effective population size (*N*_*e*_) derived from Bayesian Skygrid analysis for the corresponding years (Rambaut et al. 2018). Spearman rank correlation was employed to test the monotonic association between CUB indices and (*N*_*e*_) (Rahman et al. 2018). Multiple testing correction was performed using the Benjamini–Hochberg (BH) procedure to control the false discovery rate (FDR), with a corrected significance threshold of *α* = 0.05.

### Statistical analysis

The statistical significance of between-group differences for quantitative indicators was evaluated via normality tests performed on each dataset. Specifically, one-way analysis of variance (one-way ANOVA) was applied to data with normal distribution and homogeneity of variance, with Tukey’s test used for *post-hoc* multiple comparisons. For data that did not satisfy the normality assumption, the non-parametric Kruskal–Wallis test was adopted, followed by Dunn’s test for pairwise multiple comparisons. All statistical analyses were finished on GraphPad Prism 10.4.1, with *P* < .05 set as the uniform threshold for statistical significance.

## Results

### Phylogenetic and temporal evolutionary trajectory of IHNV

To clarify the phylogenetic relationships among IHNV genotypes, the evolutionary history of IHNV was comprehensively analysed by building a phylogenetic tree based on the whole genome (W-tree) and a phylogenetic tree based on the G gene. Four genotypes of IHNV were split into two major branches in W-tree without genotype L, and high bootstrap support for most nodes (≥95%) (Fig. 1a). The genotype U isolates formed an independent lineage located at the base of the W-tree. The genotypes J, M, and E clustered together. The group of genotype J clustered together in a single well-supported clade in IHNV. Genotypes M and E were sister taxa with 100% bootstrap support. Five genotypes of IHNV were split into two major branches in the G gene-based tree (Fig. 1b). The genotype L of IHNV isolates formed an independent lineage was located at the base of the G gene-based tree. The genotypes U, J, M, and E clustered together. Within this cluster, a subset of genotype U isolates formed a sister group with genotype J, and this clade subsequently clustered with the remaining U isolates. Two isolates of genogroup M of IHNV (GenBank: L40882.1/L40875.1) formed a sister clade with genogroup E, and the resulting clade subsequently clustered with the remaining isolates of genogroup M. The results indicated that genogroups U, J, M, and E were monophyletic in whole-genome phylogenetic tree of IHNV, and genogroups L, J, and E were monophyletic, whereas genogroup U, M were paraphyletic in G gene-based phylogenetic tree.

**Figure 1:**
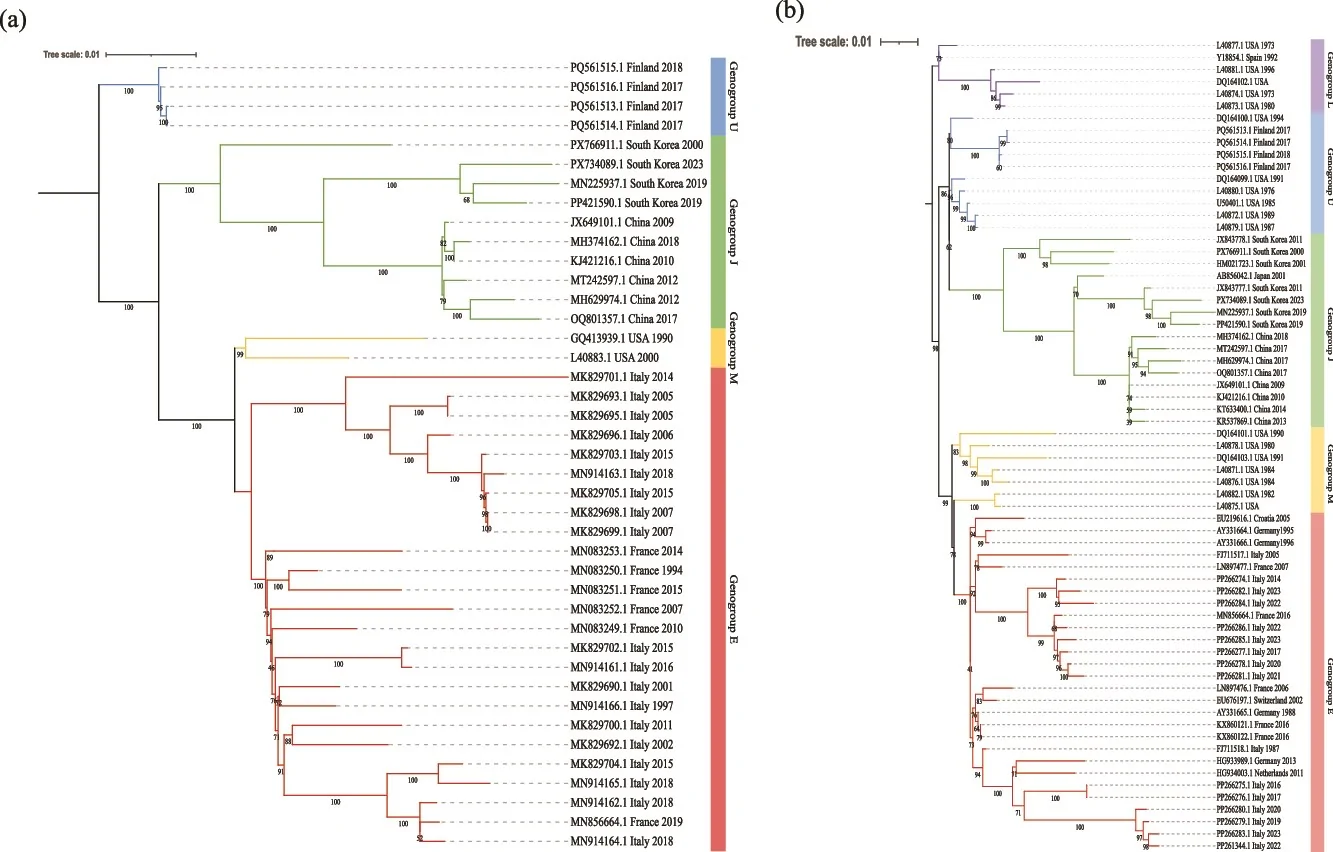
Phylogenetic and temporal evolutionary trajectory of IHNV genotypes based on complete genome (a) and G gene (b). Values at nodes indicate support percentages. The genotype of IHNV is shown to the right.

To further investigate the causes of the observed topological discrepancy, we conducted homologous recombination screening. A total of three candidate recombination events were detected in the whole-genome sequences. As shown in Table 1, only Event 1 was supported by significant *P*-values from seven algorithms and was therefore classified as a high-confidence inter-genotypic recombination event. The breakpoints of Event 1 were located in the terminal regions of the genome. No recombination breakpoints were identified within the coding sequence when the G gene open reading frame was analysed separately. The results indicated that the topological discrepancy between the W-tree and the G gene-based tree was not caused by inter-genotypic recombination.

**Table 1:** Putative recombination signals of IHNV whole-genome sequences.

Recombination event number	1	2	3
Begin	10 994	11 035	11 092
End	162	11 086	11 151
Recombinant Sequence (s)	MN225937	MN914163	OQ801357
Minor Parental Sequence (s)	MK829692	MK829692	Unknown (GQ413939)
Major Parental Sequence (s)	PP421590.1	MK829705	KJ421216
RDP	9.17E-10	NS	NS
GENECONV	3.75E-06	2.37E-05	2.95E-03
Bootscan	5.12E-10	1.20E-06	3.91E-02
Maxchi	5.15E-03	NS	NS
Chimaera	2.14E-04	NS	NS
SiScan	1.75E-02	NS	NS
3Seq	4.03E-09	1.09E-02	NS

Note: NS = not significant (*P* ≥ .05). Breakpoints indicate nucleotide coordinates in the wholegenome alignment. Event 1 represents a high-confidence recombination supported by all seven algorithms. Events 2 and 3 are tentative weak recombination signals supported by fewer than four algorithms.

To further study the evolution of IHNV genotypes, we performed Bayesian molecular-clock analysis using the G gene sequences and constructed a time-calibrated tree. As shown in Fig. 2, the IHNV lineages diverged into two clades around 1958 ce (posterior probability: 1): clade I comprised IHNV genotypes L, U, and J, and clade II comprised IHNV genotypes E and M. The genotype L of IHNV diverged around 1959 ce (posterior probability: 0.41) in clade I. The genotype U diverged around 1966 ce (posterior probability: 0.96), and genotype J diverged around 1966 ce (posterior probability: 0.28). The genotype M diverged around 1965 ce (posterior probability: 0.93) in clade II, and genotype E diverged around 1968 ce (posterior probability: 0.88) (Fig. 2). The results indicated that the divergence events of the five IHNV genotypes were concentrated in the 1950s–1970s, with genotype L representing the most ancient lineage and likely contributing substantially to the overall genetic diversity of IHNV.

**Figure 2:**
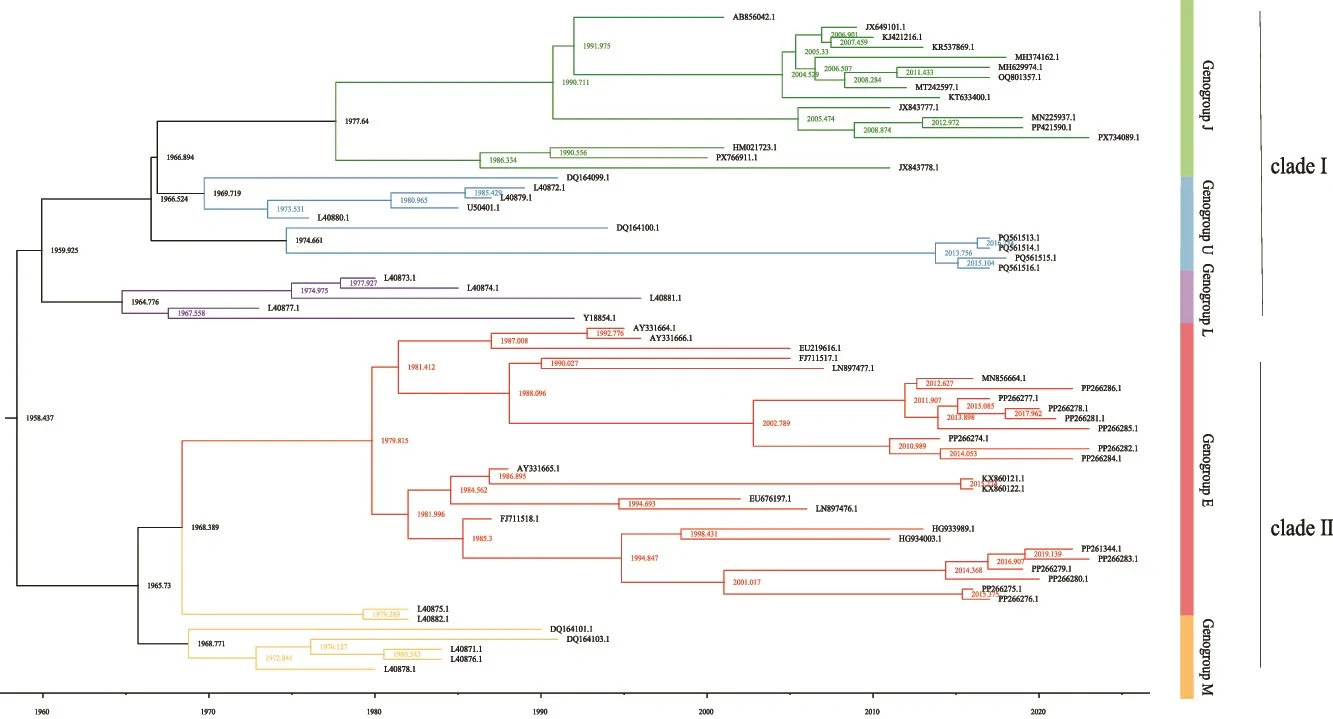
Analysis of temporal evolutionary trajectory based on IHNV G gene sequences. Values at node represent the estimated divergence year of ancestral lineages. The genotype of IHNV is shown to the right.

### Differences in codon usage across IHNV genotypes

To investigate the relationship between codon usage patterns and genotypes of IHNV, we thoroughly examined codon usage patterns by computing the RSCU for each genotype at both the whole-genome and G gene levels. At the whole-genome and G gene levels, the most frequently preferred codons were related to the encoding of Leu (CTC), Ser (TCC), Pro (CCC), and Thr (ACC) across all genotypes (RSCU > 1.6) (Fig. 3a and b). At the G gene level, additional preferred codons were identified as encoding Leu (CTG), Arg (AGG), and Gly (GGG) across all genotypes (Fig. 3b). At the whole-genome and G gene levels, the most infrequently used codons were encoding Leu (TTA/TTG), Ser (TCG), and Arg (CGT) across all genotypes (RSCU < 0.6) (Fig. 3b). At the whole-genome level, the preferred codon was associated with codon encoding Leu (CTG) in genotype U of IHNV, whereas the underrepresented codon was associated with codon encoding Arg (CGC) in genotypes J and E of IHNV (Fig. 3a). At the G gene level, the additional preferred codons were associated with Arg (AGA) and Ala (GCC) in genotype J of IHNV; the significantly avoided codons were associated with Ser (AGT) and Leu (CTA) in genotypes L, J, and U of IHNV; and the underrepresented codon was associated with Arg (CGC) in genotypes L, J, and E of IHNV (Fig. 3b). The results indicated that CUB was affected by both multiple evolutionary pressures and nucleotide composition in IHNV; the differences in CUB across the genotypes of IHNV may be reflected by the adaptive adjustments of the virus to the host environment during its evolution.

**Figure 3:**
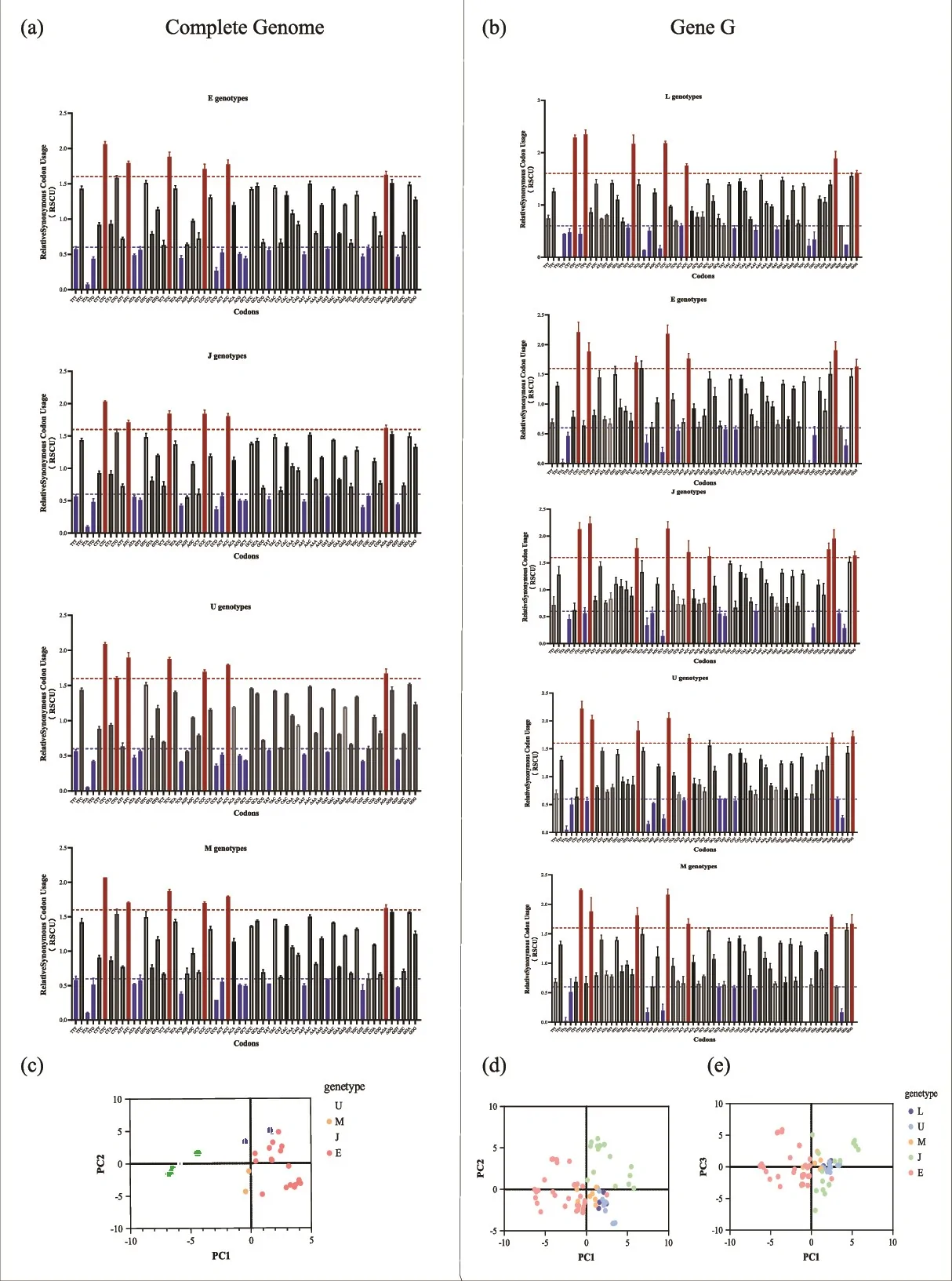
Analysis of RSCU and PCA across IHNV genotypes. (a) RSCU of whole genome in IHNV. (b) RSCU of G gene in IHNV. (c) PCA of RSCU in whole genome. (d, e) PCA of RSCU in the G gene.

To evaluate the overall link between codon usage patterns and genotypic differentiation of IHNV, we further performed PCA. PC1 and PC2 explained 40.03% of total variation (24.64% and 15.39%) in the whole genome of IHNV (Fig. 3c). The plot also revealed that while each genotype of IHNV formed distinct clusters, the genotype J of IHNV formed a single cluster, while genotypes U, M, and E of IHNV were more dispersed. The count of PC1, PC2, and PC3 explained 36.47% of variation in G gene. The PCA projection of RSCUs onto two principal axes uncovers a unique clustering pattern, and the genotypes L and U of IHNV strains are highly overlapped in the PC1 versus PC2 plot, and the genotypes L, U, and M of IHNV strains are highly overlapped in the PC1 versus PC3 plot (Fig. 3d and e), where viral strains group as per the respective genotypes. The results implied that different genotypes showing codon usage preferences may be affected by its evolutionary trajectory or adaptation to hosts in geographical distribution.

### CUB among genotypes of IHNV

Codon usage patterns result from multiple evolutionary forces, including host adaptation, natural selection, and mutational pressure. To probe into the impact of genotypic differentiation on codon usage, we thoroughly examined CUB based on whole-genome data (excluding genotype L due to missing data) and G gene sequences of IHNV. At the whole-genome level, all genotypes of IHNV demonstrated stronger usage of adenine (A) but weaker usage of thymine (T), except genotype L of IHNV, which was excluded due to incomplete genome data (Fig. 4a). Furthermore, a general inclination towards C utilization at the third codon position was evident throughout the whole genome, while the preference for T usage was comparatively weaker (Fig. 4a). This overall pattern suggests that viral genome evolution may be jointly driven by intrinsic mutational pressure during replication and the impact of natural selection. Similarly, analysis of the G gene showed that its third codon position usage frequency aligns with that of the whole genome (Fig. 4b). The results indicated that such codon usage patterns were not merely random genomic features but also reflected retained translational efficiency advantages and evolutionary adaptability in key functional genes of the virus.

**Figure 4:**
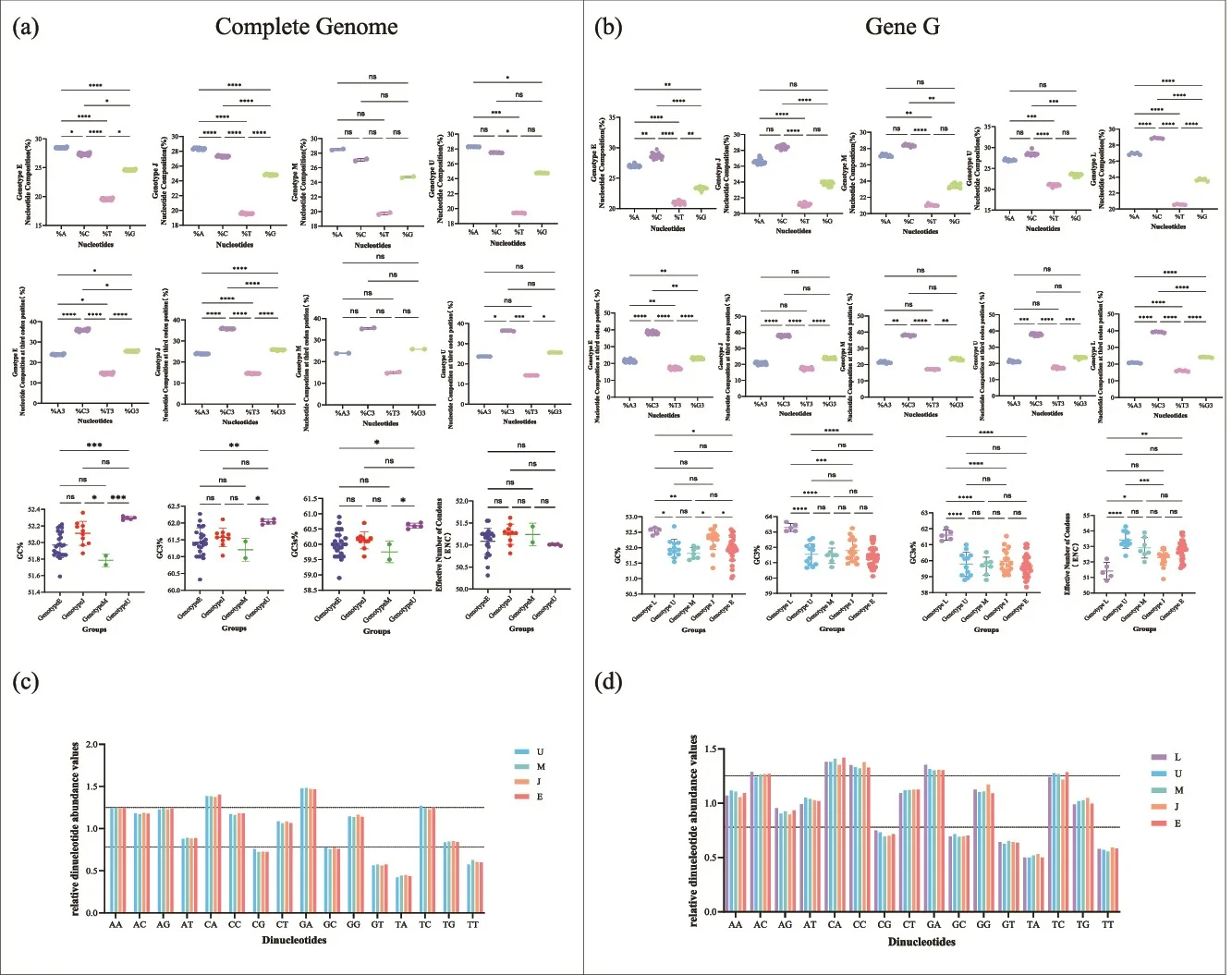
Differences in codon usage characteristics among IHNV genotypes. Analyses of codon usage characteristics based on complete genome (a) and G gene (b). Analyses of relative abundance of dinucleotides based on complete genome (c) and G gene (d). ^*^P < .05, ^**^P < .01, ^***^P < .001, ^****^P < .0001.

At the whole-genome level, the GC content of all IHNV genotypes varied from 51.5% to 52.4% and the ENC varied from 50 to 52 (Fig. 4a). At the G gene level, the GC content of all IHNV genotypes ranged from 50.5% to 53% and the ENC ranged from 50 to 55 (Fig. 4b). Furthermore, at the whole-genome and G gene levels, the GC3s content of all genotypes were significantly higher than their respective average GC contents (Fig. 4). For inter-genotype differences, at the whole-genome level, the average levels of GC, GC3, and GC3s of genotype U were higher than other genotypes of IHNV, while the mean ENC of genotype U was lower than other genotypes of IHNV (Fig. 4a). At the G gene level, the GC, GC3, and GC3s of genotype L were significantly higher than other genotypes of IHNV, while the ENC of genotype L was the lowest in all genotypes of IHNV (Fig. 4b). The results indicated that this phenomenon arose from stronger natural selection pressure or adaptation to specific evolutionary constraints. The moderately high GC content of IHNV implied the key adaptive characteristics of the virus to the host, which helps improve translation efficiency and achieve immune evasion.

Dinucleotide relative abundance analysis revealed that dinucleotide usage preferences were largely consistent across all genotypes of IHNV (Fig. 4c). At both whole-genome and G gene levels of analysis, CpG, GpC, GpT, TpT, and TpA were markedly underrepresented across all genotypes of IHNV, while CpA, GpA, and TpC were overrepresented. Additionally, CpC was significantly overabundant across G gene of IHNV (Fig. 4d). The results indicated that IHNV reduces host-recognizable or unstable dinucleotides such as CpG and TpA, while accumulating mutant products like CpA and GpA. The results demonstrated that the virus was driven by both host-induced methylation/deamination mutations and immune evasion selection during evolution.

To evaluate whether the observed dinucleotide bias was affected by reading frame constraints, we performed a Monte Carlo resampling analysis. As shown in Table 2, the *ρ*^MC^ values of dinucleotides CpG, GpC, GpT, TpT, and TpA were all significantly less than 1, with all *P* values < .001 in both the whole-genome and G gene datasets. The results confirmed that these dinucleotides were subjected to strong selective pressure during the evolution of the IHNV genome, exhibited a pronounced usage avoidance signature. In contrast, the overrepresented dinucleotides including TpC, TpG, CpT, CpA, GpA, and GpG exhibited *ρ*^MC^ values close to or greater than 1, with no statistical significance (*P* ≥ .999). The results indicated that the elevated abundance of these overrepresented dinucleotides was a natural consequence of genomic amino acid composition and codon usage, rather than a product of additional directional selection.

**Table 2:** Dinucleotide composition analysis of IHNV complete genome and G gene sequences.

Dinucleotide	Whole genome *ρ* ^MC^	Whole genome *P*-value	G gene *ρ* ^MC^	G gene *P*-value
TpA	0.3473	<.001	0.3451	<.001
TpT	0.6605	<.001	0.4914	<.001
GpC	0.6657	<.001	0.6487	<.001
CpG	0.7281	<.001	0.7486	<.001
GpT	0.8584	<.001	0.7723	<.001
ApA	0.9117	<.001	0.9249	<.001
CpC	0.9273	<.001	0.8487	<.001
ApC	1.0184	NS	0.9588	.017
ApT	1.0231	NS	1.0687	NS
ApG	1.0591	NS	1.0548	NS
GpG	1.1316	NS	1.0404	NS
GpA	1.1498	NS	1.2945	NS
CpA	1.2095	NS	1.1427	NS
CpT	1.2191	NS	1.3448	NS
TpG	1.3014	NS	1.4273	NS
TpC	1.6423	NS	1.6675	NS

Note: *ρ*^MC^ > 1 indicates overrepresentation, *ρ*^MC^ < 1 indicates underrepresentation; *P* < .05 indicates significant deviation from expected frequency; NS = not significant (*P* ≥ .05)

### Evolutionary forces framing CUB in IHNV genotypes

To evaluate the forces shaping CUB across IHNV genotypes, the relative contributions of natural selection and mutational pressure were quantified using PR2-bias, ENC-plot, and neutrality analyses. Codon usage for every genotype departs from the PR2-plot origin (0.5, 0.5) to varying extents in both the G gene and the whole genome (Fig. 5), indicating contributions from both mutation and selection. Moreover, all data points fall below the expected ENC curve (Fig. 5), a pattern that implicates selection pressure as the dominant force driving codon usage in these genotypes.

**Figure 5:**
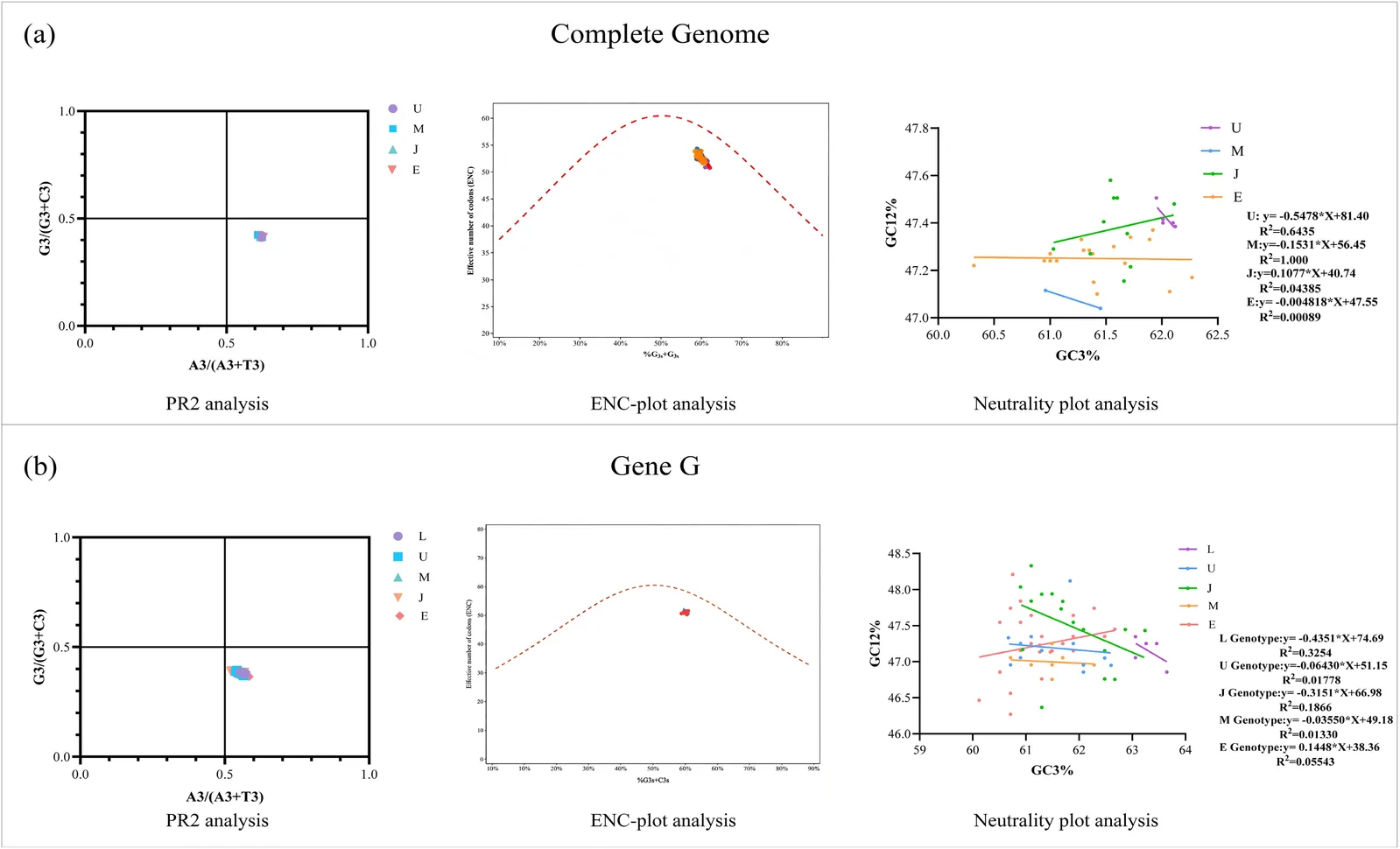
Analysis of evolutionary forces driving CUB in IHNV genotypes based on complete genome (a) and G gene (b).

The relative impacts of selective and mutational pressures on CUB were quantified via neutrality analysis, which compares the link between GC12% and GC3% to reveal the balance of evolutionary forces. GC3 ranged from 60.0% to 62.5%, while GC12 varied from 47.0% to 47.8% based on the whole-genome of IHNV (Fig. 5a). The genotypes U, J, and E of IHNV yielded a slope (b) of −0.5478 (regression equation: y = −0.5478x + 81.40, coefficient of determination *R*^2^ = 0.6435, *P* = .1024, 95% CI of slope: −1.297 to 0.2013), 0.1077 (y = 0.1077x + 40.74, *R*^2^ = 0.04385, *P* = .5615, 95% CI of slope: −0.3023 to 0.5178), and −0.004818 (y = −0.004818x + 47.55, *R*^2^ = 0.00089, *P* = .9033, 95% CI of slope: −0.08722 to 0.07758), respectively. The genotype M yielded *R*^2^ = 1.000 due to collinearity of only two data points with no statistical significance (Fig. 5a). For the whole-genome datasets of IHNV, the selection contribution metric values for genotypes U, J, and E were 45.22%, 89.23%, and 99.52%, respectively. The absolute values of the regression slopes for the most genotypes of IHNV were substantially less than 1, except for genotype M. GC3 varied from 59.0% to 64.0%, while GC12 ranged from 46.0% to 48.5% as per the G gene of IHNV (Fig. 5b). The genotypes L, J, U, M, and E of IHNV yielded a slope (b) of −0.4351 (y = −0.4351x + 74.69, *R*^2^ = 0.3254, *P* = .3153, 95% CI of slope: −1.586 to 0.7159), −0.3151 (y = −0.3151x + 66.98, *R*^2^ = 0.1866, *P* = .0833, 95% CI of slope: −0.6771 to 0.04691), −0.06430 (y = −0.06430x + 51.15, *R*^2^ = 0.01778, *P* = .6795, 95% CI of slope: −0.4010 to 0.2724), −0.03550 (y = −0.03550x + 49.18, *R*^2^ = 0.01330, *P* = .8055, 95% CI of slope: −0.3871 to 0.3161), and 0.1448 (y = 0.1448x + 38.36, *R*^2^ = 0.05543, *P* = .1801, 95% CI of slope: −0.07046 to 0.3602), respectively. For the G gene datasets of IHNV, the selection contribution metric values for genotypes L, J, U, M, and E were 56.49%, 68.49%, 93.57%, 96.45%, and 85.52%, respectively. The results indicated that selection contributed substantially more than mutation to shaping codon usage patterns in IHNV, and selection played a dominant role in driving CUB in IHNV.

The absolute values of the regression slopes for all genotypes were all substantially less than 1 according to the G gene of IHNV. The results suggested that for every 1% increment in GC3, the maximum change in GC12 was only about 0.55% and 0.44% based on the whole-genome and G gene of IHNV, respectively. Meanwhile, all genotypes presented relatively low *R*^2^, demonstrating that mutation pressure could only explain a small proportion of the variation in codon usage. The vast majority of data points clearly deviated from the diagonal representing uniform mutation pressure. The results indicated the dominant role of natural selection in the evolution of codon usage patterns in both the whole genome and G gene of IHNV, while mutation pressure exerted a limited overall effect. The results from PR2, ENC, and neutrality analyses jointly indicated that selection pressure mainly promoted the evolutionary trajectory whole genome and G gene in IHNV, while mutational pressure had a secondary effect. This highlights the central significance of functional optimization and host-specific adaptation in framing codon usage preferences across IHNV genotypes.

### Host adaptation and codon usage optimization of IHNV genotypes

To explore whether codon differences result in variations in host adaptation between genotypes, we used CAI, RCDI, and SiD to study whether codon differences can change host adaptation between genotypes. IHNV is the most adaptable to salmonids (*O. tshawytscha, O. mykiss*), owing to higher CAI, lower RCDI, and lower SiD than non-salmonid (*E. lucius*) (Fig. 6). At the whole-genome level, all three indices (CAI, RCDI, and SiD) showed highly significant differences in all pairwise comparisons among the three host species (*P* < .0001). At the G gene level, both salmonid species exhibited highly significant differences from *E. lucius* across all indices (*P* < .0001), between the two salmonid hosts, significant differences were detected for CAI (*P* = .0005) and SiD (*P =* .0141), but not in RCDI (*P* > .05) (Fig. 6). The results indicated that fishes from the family Salmonidae act as a main host for IHNV, as the virus displayed an optimized codon usage pattern for effective translation within this species. For inter-genotype pairwise comparisons under each host background, CAI, RCDI, and SiD values were highly similar across all IHNV genotypes, and no statistically significant differences in CAI, RCDI, or SiD were observed across all IHNV genotypes for either the complete genome and G gene dataset (*P* > .05). There were no significant differences in host adaptation in different genotypes IHNV (Fig. 6), revealing a conserved host preference across genotypes. The consistent adaptation patterns observed at both the G gene and whole-genome levels indicated that the virus may be under strong selective pressure to maintain synergistic optimization with the translational machinery of its primary hosts.

**Figure 6:**
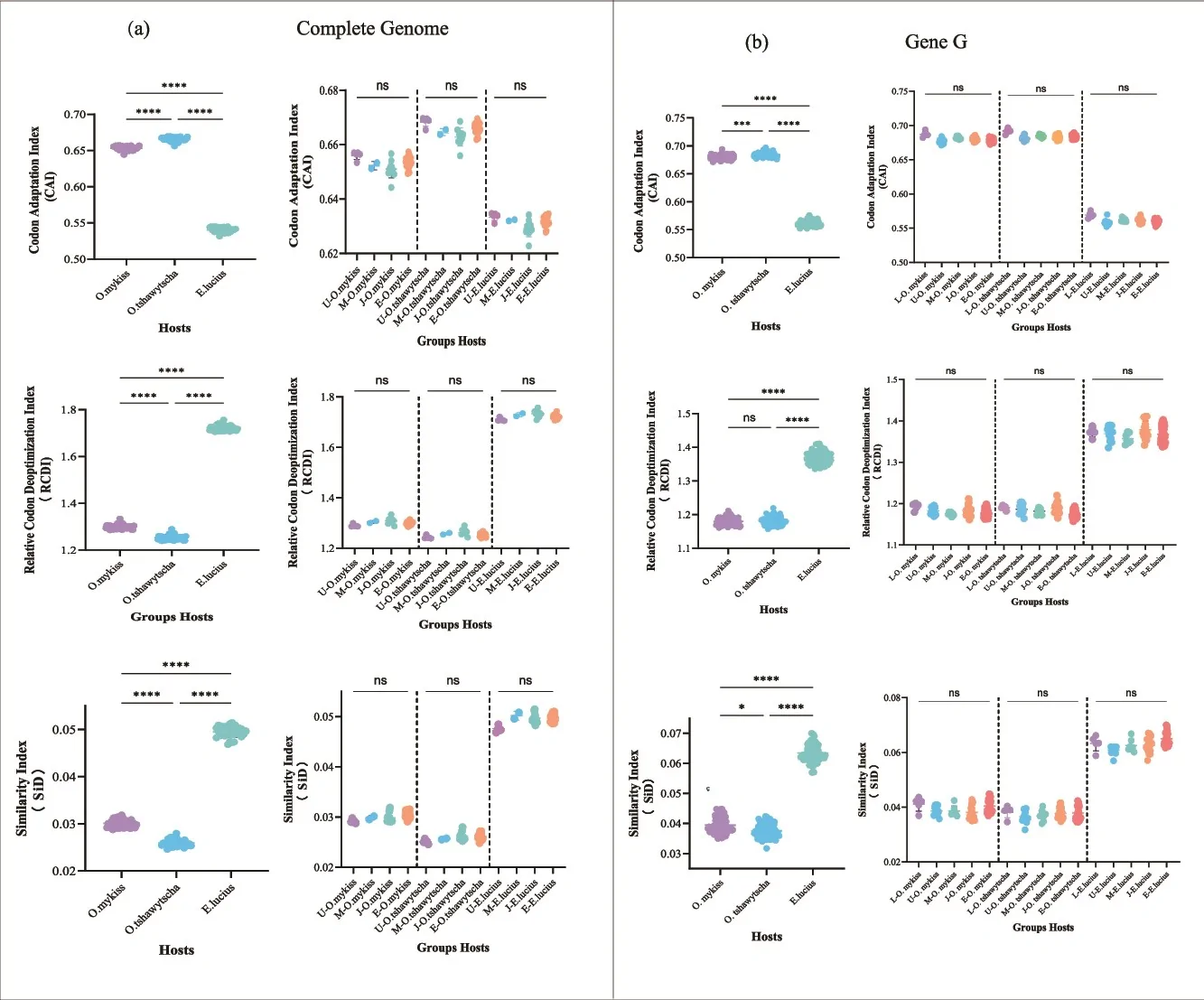
Host adaptation analysis of IHNV genotypes based on complete genome (a) and G gene (b). ^*^P < .05, ^**^P < .01, ^***^P < .001, ^****^P < .0001.

### Population expansion in context of codon usage

To examine IHNV population expansion in relation to CUB, we reconstructed the demographic history of IHNV overall and for each of the five genotypes using Bayesian skyline analysis. First, we tested the temporal signal of each dataset with TempEst. All genotype datasets exhibited extremely strong temporal structure (*R*^2^_J = 1, *R*^2^_L = 1, *R*^2^_M = 1, *R*^2^_U = 1, *R*^2^_E = 1, Fig. 7), indicating a high correlation between sampling time and genetic distance across genotype sequences. This result provided ideal conditions for high-confidence inference of historical population dynamics. The Bayesian skyline analysis further revealed the trajectory of population size changes in IHNV under specific codon adaptation states. On the general level, the IHNV population began to grow rapidly from 1955, showed a stable trend between 1985 and 2000, and then began to decline after 2000 (Fig. 7). At the individual level, different populations grew and contracted. Genotype E had clear population expansion starting from 1985, a peak around 1992 and a stable until sharp decline and contraction around 2010, and then a steady expansion trend around 2015. Genotype J showed a stable trend from 1985 to 2005, clear and rapid contraction between 2005 and 2008, a sharp rebound, a peak around 2013, and a relatively stable population size. Genotype M experienced slow population growth around 1980, after which the population size stabilized. Genotype U experienced continuous and significant population growth from 1975 to 1987, after which growth slowed and stabilized, and showed a population contraction after 2010. Genotype L showed the first signs of population activity. Slow but continuous expansion started around 1970, then significant population expansion between 1975 to 1980, peaking around 1980 and stabilizing (Fig. 7). The results showed that different genotypes of IHNV had different population dynamics, which may be due to epidemiological dynamics, host adaptation, and genetic constraints.

**Figure 7:**
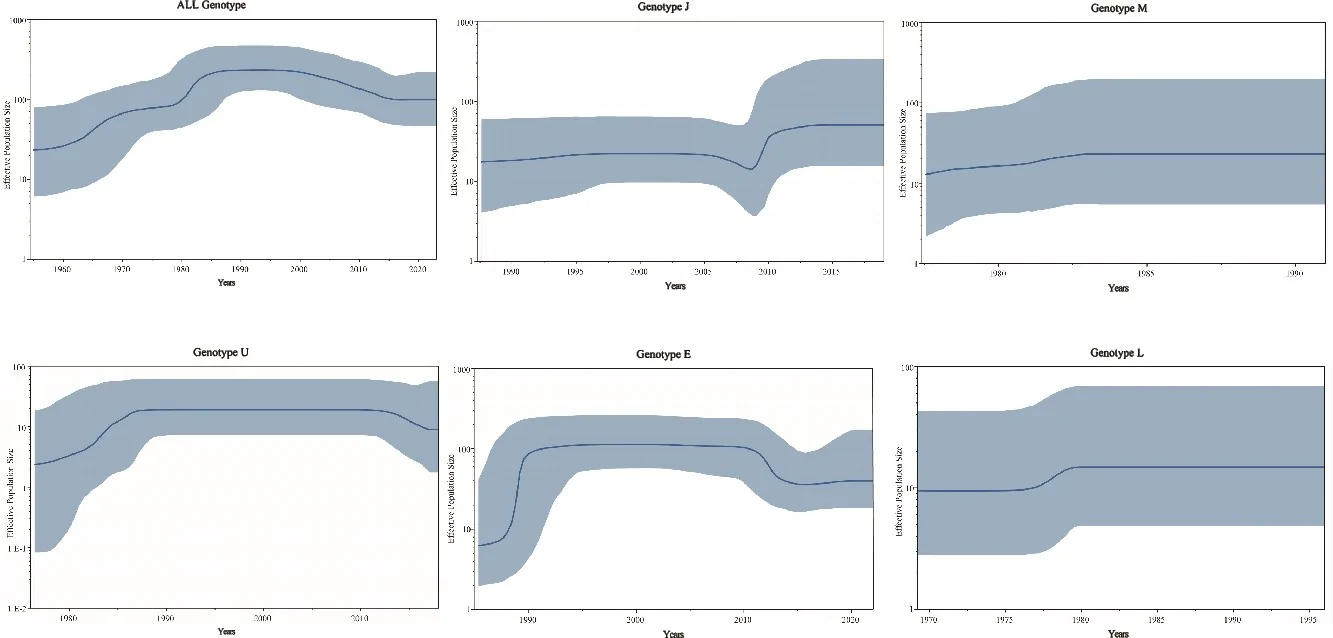
Population expansion of each genotype in the context of codon usage. The shaded area denotes the 95% HPD interval for the product of generation time and effective population size (*Ne* × τ); the solid line indicates the median.

### Results of the association analysis

To explore the potential association between population dynamics and CUB of IHNV, correlation analyses were individually performed for each IHNV genotype. As shown in Table 3, the Spearman correlation coefficients (*r*_s_) for GC3s ranged from −0.013 (Genotype J) to 0.5 (Genotype M), while those for ENC ranged from −0.659 (Genotype U) to 0.783 (Genotype M). Although some individual *r*_s_ values appeared moderate to strong, none of the correlations remained significant after correction for multiple testing (*q* = 0.322–0.968). The results indicated that codon usage variation in each IHNV genotype did not track short-term fluctuations in population size.

**Table 3:** Spearman rank correlation between codon usage bias and effective population size in each IHNV genotype.

Test group	Spearman (*r* _s_)	Annual sample size	FDR-corrected *q*-value (BH method)	Significance (adjusted α = 0.05)
Genotype J – GC3s	−0.013	15	0.968	NS
Genotype J – ENC	0.56	15	0.322	NS
Genotype U – GC3s	0.479	8	0.582	NS
Genotype U – ENC	−0.659	8	0.462	NS
Genotype L – GC3s	0.354	5	0.968	NS
Genotype L – ENC	−0.354	5	0.968	NS
Genotype M – GC3s	0.5	5	0.968	NS
Genotype M – ENC	0.783	5	0.582	NS
Genotype E – GC3s	0.142	17	0.968	NS
Genotype E – ENC	0.011	17	0.968	NS

## Discussion

In this communication, we reported details of CUB in different genotypes of IHNV as well as host adaptation, evolution, and population dynamics. Our comparison shows that natural selection plays a dominant role in IHNV CUB and that glycoprotein G emerges as one of the major adaptive genes in genotypes. While IHNV adapts to its primary salmonid hosts, different genotypes have different evolutionary trajectories and population histories, possibly depending on their codon usage profiles.

The phylogenetic analyses revealed a discrepancy between the whole-genome (W-tree) and G gene-based trees (Fig. 1). The genotypes U and L of IHNV were positioned at the base of the tree in the W-tree and G gene-based trees, respectively, while the remaining genotypes of IHNV formed a well-supported clade. The topological discrepancy was most likely attributed to the absence of genotype L sequences in the whole-genome dataset and the use of different outgroup configurations, as genotype L is widely recognized as the most ancestral IHNV lineage originating from the Pacific Northwest of North America (Bellec et al. 2017). Furthermore, the G gene serves as the primary target of host neutralizing antibodies (Huang et al. 1994) and is subjected to intense immune-driven directional selection. This selective pressure may accelerate its evolutionary rate relative to the rest of the genome, thereby resulting in topological incongruence between the G gene-based trees and W-tree. Both phylogenetic trees revealed distinct geographic clustering. Strains within genotype J were all isolated from East Asia, genotype M strains were mainly isolated from North America, genotype E strains were isolated from Europe, while genotype U of IHNV exhibited a wide transregional distribution. Our temporal analysis further pinpoints the major genotypic diversification of IHNV to the period between the 1950s and 1970s. This era coincides with the intensification and global expansion of rainbow trout aquaculture, suggesting that anthropogenic factors, such as fish movement and high-density farming, may have provided the ecological catalyst for viral radiation and adaptation, a hypothesis supported by epidemiological models of IHNV spread (Garver et al. 2003, Kurath et al. 2003).

The analysis of RSCU confirmed a core set of preferred and avoided codons across all genotypes of IHNV, superimposed on this common framework were clear genotype-specific preferences. This result indicates that different lineages have fine-tuned their CUB, possibly as a result of adaptation to specific ecological niches, host populations, or in response to unique historical selection bottlenecks (He et al. 2013).

PCA of RSCU revealed that genotype J of IHNV formed an independent and clustered group based on whole-genome, reflecting relatively conserved codon usage across the genome. Genotypes U, M, and E showed more dispersed distributions, indicating greater divergence in CUB during evolution. The PCA clearly distinguished all genotypes of IHNV based on G gene, with partial overlap observed among the North American-derived genotypes L, U, and M. Notably, there was geographically associated subpopulation differentiation within genotype J. The IHNV CUB is non-random and jointly shaped by genotypic divergence, geographical origin, and host adaptation (Boudinot et al. 2017), providing complementary insights into the evolution trajectory and regional adaptation of IHNV. A central finding of this study is the clear dominance of natural selection over mutational pressure in framing IHNV CUB. This conclusion is robustly advocated by multiple lines of evidence. ENC-plot analysis shows that the distribution of all genotypes below the expected ENC curve is a classic indicator that selection, instead of mutational bias alone, is the major force constraining codon choice (Deb et al. 2020, Dilucca et al. 2020). Neutrality plot analysis demonstrates that variations in GC3 (often influenced by mutation) are poorly correlated with variations in GC12 (more constrained by protein function). This indicates that selection pressure on the amino acid sequence effectively decouples the evolution of the first two codon positions from the third, allowing the latter to be optimized for translational efficiency independent of mere mutational trends (Sueoka 1988).

The GC contents of whole-genome and G gene were moderately high in IHNV, and GC3s contents of whole-genome and G gene were significantly higher than average GC contents of each genotype of IHNV. However, studies on SVCV and CyHV-3 reported significant GC12-GC3 correlations and attributed codon usage primarily to mutational pressure rather than translational selection (Ma et al. 2014, Ma et al. 2015). Notably, although CyHV-3 also prefers G/C-ending codons, this preference was linked to genome-wide mutational pressure (Ma et al. 2015). In contrast, this GC3 preference in IHNV likely reflects adaptive tuning of the virus to host tRNA pools, where translational selection favours G/C-ending codons, enhancing translation speed and accuracy, leading to marked GC enrichment at the third codon position and accuracy and consequently elevating the overall genomic GC level (Ventoso 2025). The dinucleotide relative abundance analysis revealed that CpG, GpC, GpT, TpT, and TpA at both whole-genome and G gene levels were markedly underrepresented across all genotypes of IHNV, while CpA, GpA, and TpC were overrepresented. Additionally, CpC was significantly overabundant across G gene of IHNV (Fig. 4c and d). Reportedly, TpA and UpA are usually slightly abundant in the genomes of DNA and RNA viruses, which can enhance nucleic acid sequence stability (Breslauer et al. 1986, Beutler et al. 1989). The scarcity of CpG in viruses has been linked to immune evasion characteristic (Jimenez-Baranda et al. 2011), by reducing dinucleotides such as CpG and TpA, which are easily recognized by the host or unstable, and accumulating mutation products like CpA and GpA. IHNV appears to be jointly driven by host-induced methylation/deamination mutational pressure and host immune evasion selection during evolution. This reflects the adaptation of IHNV to the host immune environment through the regulation of dinucleotide composition while maintaining replication efficiency.

The IHNV naturally infect salmonids such as *O. mykiss* and *O. tshawytscha*. There have also been reports of non-salmonid hosts being infected with IHNV, such as *E. lucius* (Cabon et al. 2020). The all IHNV genotypes show higher adaptation (higher CAI, lower RCDI/SiD) to the two salmonid hosts, *O. mykiss* and *O. tshawytscha*, compared to *E. lucius*. Crucially, no significant differences in this adaptation pattern were observed among genotypes. It suggests that the fundamental codon usage optimization of IHNV is for the salmonid translational environment, representing a deep evolutionary adaptation that precedes and underlies the more recent genotypic diversification. This salmonid-optimized CUB may represent a major genetic constraint that limits the virus’s ability to jump efficiently to non-salmonid hosts (Tello et al. 2013).

The population growths of most genotypes starting in the 1970s and 1980s are consistent with the global aquaculture boom (Asche et al. 2008). Genotype U is fast expanding and could be linked to a balance between host adaptation and evolutionary plasticity. The dramatic contraction and rebound of genotype J could represent epidemiological dynamics in a particular host population or region, possibly linked to vaccination campaigns or changes in farming practices. The distinct patterns could be linked to founder effects, differences in transmission rate in various aquaculture systems, and possibly variations in host adaptation efficiency as a consequence of their CUB profiles. However, the lack of correlation between CUB metrics and effective population size (*q* > 0.05) suggests that these selective constraints operate on longer evolutionary timescales and are not tightly coupled with recent demographic fluctuations captured by the Skyline analysis (Sharma et al. 2024). This discrepancy highlights the need to incorporate codon usage data with detailed epidemiological records to disentangle correlation from causation.

Our results are consistent with and extend the current understanding of viral codon use and evolution. The leading role of natural selection in framing CUB is consistent with studies on other single-stranded RNA viruses with complex life cycles and host interactions, such as influenza (Zhang et al. 2019), coronaviruses (Nyayanit et al. 2020), and rhabdoviruses (Sharma et al. 2024) and with some DNA viruses or bacteriophages where mutation pressure plays a larger role, suggesting importance of replication strategy and host environment (Chithambaram et al. 2014). Gene-specific evolution: the distinct evolutionary behaviour of IHNV G gene is consistent with observations observed in other viruses with prominent surface proteins (e.g. HIV-1 env, HCV E1/E2) (Boonchawalit et al. 2011). CpG suppression and immunity escape hypothesis: the observed underrepresented CpG dinucleotides is a common evolutionary feature across different vertebrate viruses (fish rhabdoviruses, human herpesviruses, Retroviruses) (Chen et al. 2001, Takata et al. 2017). Host-specific adaptation: the clear codon adaptation to salmonids (not a general piscine host pattern) gives a molecular basis for the host range of IHNV. This is similar to studies show that codon adaptation of human-specific viruses is often optimized for human cells than for other mammals (Bahir et al. 2009).

Reliance on standard codon usage metrics (CAI, RCDI, SID) provides only an indirect assessment of translational fitness; direct integration of tRNA-Seq data from infected *O. mykiss* tissues would yield greater accuracy (Pinkard et al. 2020). Experimental validation of G-gene mutants via reverse genetics is lacking, as are paired comparisons between virulent and attenuated strains. Moreover, the proposed link between codon usage and epidemiological parameters (e.g. virulence, transmission) remains untested, and non-coding/intergenic regulatory regions were omitted. Beyond these methodological extensions, the observed biases could be exploited for synonymous codon deoptimization, offering a rational strategy for developing live-attenuated IHNV vaccines (Coleman et al. 2008).

## Conclusions

This study provided, for the first time, comprehensive characterization of codon usage patterns, evolutionary forces, host adaptation, and population dynamics across the five genotypes of IHNV. The results revealed that divergence events among IHNV genotypes were predominantly concentrated between the 1950s and 1970s, with genotype L of IHNV representing the most ancestral lineage. Codon usage of IHNV exhibited both genotype-specific features and overall conservation. Natural selection emerged as the primary driving force shaping codon usage bias of IHNV. All IHNV genotypes shown markedly higher codon adaptation to salmonid fishes than to non-salmonid hosts. Furthermore, the demographic histories of individual genotypes of IHNV differ significantly. Collectively, these results extended current understanding of the molecular mechanisms underlying adaptive evolution in IHNV and offer a theoretical foundation for elucidating the evolutionary trajectories of aquaculture-associated viruses.

## Supplementary Material

Supplementary_materials_veag062

## Data Availability

All data analysed here are publicly available from the NCBI database. The complete lists of accession numbers for both whole genomes and G gene sequences are shown in Supplementary Table S1 and S2.
