# Supplementary material for: Comparative analysis of codon usage bias revealed natural selection-driven evolution and host adaptation of infectious haematopoietic necrosis virus
Source: Virus Evol. 2026 Sep 21;12(1):veag062. doi: 10.1093/ve/veag062 (PMC13652644; doi:10.1093/ve/veag062)
Supplement: Supplementary file 1 [file supplementary_materials_veag062.zip › Supplementary_materials_veag062_Table S2.docx]

Supplementary Table S2. Accession numbers of the IHNV G gene sequences used in this study.

| No. | Strain Name | Accession Number | Genotype | Geographic Origin | Host |
| --- | --- | --- | --- | --- | --- |
| 1 | SRCV | L40881 | L | USA | / |
| 2 | FR0031 | DQ164102 | L | USA | Oncorhynchus tshawytscha |
| 3 | Co1-80 | L40873 | L | USA | / |
| 4 | Co1-85 | L40874 | L | USA | / |
| 5 | LR-73 | L40877 | L | USA | / |
| 6 | S46 | Y18854 | L | Spain | Oncorhynchus mykiss |
| 7 | FF030 | DQ164099 | U | USA | sockeye salmon |
| 8 | RB-76 | L40880 | U | USA | / |
| 9 | LWS-87 | L40879 | U | USA | / |
| 10 | Carson-89 | L40872 | U | USA | Spring Chinook salmon |
| 11 | Round Butte 1 | U50401 | U | USA | / |
| 12 | BLK94 | DQ164100 | U | USA | sockeye salmon |
| 13 | 1248/17 | PQ561516.1 | U | Finland | Oncorhynchus mykiss |
| 14 | 16/18 | PQ561515.1 | U | Finland | Oncorhynchus mykiss |
| 15 | 967/17 | PQ561514.1 | U | Finland | Oncorhynchus mykiss |
| 16 | 820/17 | PQ561513.1 | U | Finland | Oncorhynchus mykiss |
| 17 | FF030-91 | DQ164103 | M | USA | / |
| 18 | LR-80 | L40878 | M | USA | / |
| 19 | HO-7 | L40876 | M | USA | / |
| 20 | 193-110 | L40871 | M | USA | / |
| 21 | 220-90 | DQ164101 | M | USA | Oncorhynchus mykiss |
| 22 | WRAC | L40882 | M | USA | ainbow trou |
| 23 | CST-82 | L40875.1 | M | USA | Oncorhynchus mykiss |
| 24 | PcOj11 | JX843778 | JRT | South Korea | / |
| 25 | RtWanJu09 | HM021723 | JRT | South Korea | / |
| 26 | PCKW11 | JX843777 | JRT | South Korea | / |
| 27 | TV0026 | AB856042 | JRT | Japan | Oncorhynchus mykiss |
| 28 | CJ-13 | KR537869 | JRT | China | Oncorhynchus mykiss |
| 29 | GS2014 | KT633400 | JRT | China | Oncorhynchus mykiss |
| 30 | SCCD | OQ801357 | JRT | China | Oncorhynchus mykiss |
| 31 | Ch20101008 | KJ421216 | JRT | China | Salvelinus fontinalis |
| 32 | KNU19 | PP421590 | JRT | South Korea | steelhead |
| 33 | Sn1203 | MT242597 | JRT | China | Oncorhynchus mykiss |
| 34 | SNUV1 | MN225937 | JRT | South Korea | Oncorhynchus mykiss |
| 35 | BJSY | MH374162 | JRT | China | Oncorhynchus mykiss |
| 36 | SX1704 | MH629974 | JRT | China | Oncorhynchus mykiss |
| 37 | HLJ-09 | JX649101 | JRT | China | / |
| 38 | RTCJ23 | PX734089.1 | JRT | South Korea | Oncorhynchus mykiss |
| 39 | RTJE00 | PX766911.1 | JRT | Jecheon | Oncorhynchus mykiss |
| 40 | IHNVN1G2 | KX860122 | E | France | / |
| 41 | IHNVN3G4 | KX860121 | E | France | Trout |
| 42 | FSK/88 | AY331665 | E | Germany | / |
| 43 | 1I0 | FJ711518 | E | Italy | Oncorhynchus mykiss |
| 44 | FSVI100/96 | AY331666 | E | Germany | / |
| 45 | FS62/95 | AY331664 | E | Germany | / |
| 46 | CH-118-02 | EU676197 | E | Switzerland | / |
| 47 | I21-11NL | HG934003 | E | Netherlands | Oncorhynchus mykiss |
| 48 | I07-13bw | HG933989 | E | Germany | / |
| 49 | i742-05 | FJ711517 | E | Italy | Oncorhynchus mykiss |
| 50 | Cro/05 | EU219616 | E | Croatia | / |
| 51 | Fft121-07h | LN897477 | E | France | / |
| 52 | F317630-06 | LN897476 | E | France | / |
| 53 | BG/67-1/Jan22 | PP261344 | E | Italy | / |
| 54 | BZ/72/Mar14 | PP266274 | E | Italy | / |
| 55 | TN/41/Jan16 | PP266275 | E | Italy | / |
| 56 | TN/126/Feb17 | PP266277 | E | Italy | / |
| 57 | TN/138/Feb17 | PP266276 | E | Italy | / |
| 58 | TN/21-2/Jan20 | PP266278 | E | Italy | / |
| 59 | TN/890-1/Nov19 | PP266279 | E | Italy | / |
| 60 | TN/82/Feb20 | PP266280 | E | Italy | / |
| 61 | TN/13-2/Jan21 | PP266281 | E | Italy | / |
| 62 | TN/1090-4/Nov23 | PP266282 | E | Italy | / |
| 63 | TN/200-2/Mar2023 | PP266283 | E | Italy | / |
| 64 | BZ/1118-4/Nov23 | PP266285 | E | Italy | / |
| 65 | TN/117/Feb22 | PP266284 | E | Italy | / |
| 66 | BZ/910-4/Oct22 | PP266286 | E | Italy | / |
| 67 | OO121.8 | MN856664 | E | France | / |
