# Supplementary material for: Comparative analysis of codon usage bias revealed natural selection-driven evolution and host adaptation of infectious haematopoietic necrosis virus
Source: Virus Evol. 2026 Sep 21;12(1):veag062. doi: 10.1093/ve/veag062 (PMC13652644; doi:10.1093/ve/veag062)
Supplement: Supplementary file 1 [file supplementary_materials_veag062.zip › Supplementary_materials_veag062_Table S1.docx]

Supplementary Table S1. Accession numbers of the IHNV complete genome sequences used in this study.

| No. | Strain Name | Accession Number | Genotype | Geographic Origin | Host |
| --- | --- | --- | --- | --- | --- |
| 1 | WRAC | L40883 | M | USA | Oncorhynchus tshawytscha |
| 2 | 220-90 | HM461966 | M | USA | rainbow trout |
| 3 | SCCD | OQ801357.1 | JRT | China | rainbow trout |
| 4 | Ch20101008 | KJ421216.1 | JRT | China | Salvelinus fontinalis |
| 5 | KNU19 | PP421590.1 | JRT | South Korea | rainbow trout |
| 6 | Sn1203 | MT242597.1 | JRT | China | rainbow trout |
| 7 | SNUV1 | MN225937.1 | JRT | South Korea | rainbow trout |
| 8 | BJSY | MH374162.1 | JRT | China | rainbow trout |
| 9 | SX1704 | MH629974.1 | JRT | China | rainbow trout |
| 10 | HLJ-09 | JX649101.1 | JRT | China | rainbow trout |
| 11 | RTCJ23 | PX734089.1 | JRT | South Korea | Oncorhynchus mykiss |
| 12 | RTJE00 | PX766911.1 | JRT | South Korea | Oncorhynchus mykiss |
| 13 | 1248/17 | PQ561516.1 | U | Finland | Oncorhynchus mykiss |
| 14 | 16/18 | PQ561515.1 | U | Finland | Oncorhynchus mykiss |
| 15 | 967/17 | PQ561514.1 | U | Finland | Oncorhynchus mykiss |
| 16 | 820/17 | PQ561513.1 | U | Finland | Oncorhynchus mykiss |
| 17 | VP0614.8 | MN083253.1 | E | France | rainbow trout |
| 18 | T00121 | MN083252.1 | E | France | rainbow trout |
| 19 | NN108 | MN083251.1 | E | France | rainbow trout |
| 20 | 23/94 | MN083250.1 | E | France | rainbow trout |
| 21 | 3Lot1 | MN083249.1 | E | France | rainbow trout |
| 22 | OO121.8 | MN856664.1 | E | France | Esox lucius |
| 23 | TN/224/Mar18 | MN914165.1 | E | Italy | rainbow trout |
| 24 | TN/459/Sep97 | MN914166.1 | E | Italy | rainbow trout |
| 25 | TN/223/Mar18 | MN914164.1 | E | Italy | rainbow trout |
| 26 | TN/220/Mar18 | MN914163.1 | E | Italy | rainbow trout |
| 27 | TN/91/Feb19 | MN914162.1 | E | Italy | rainbow trout |
| 28 | TN/72/Feb16 | MN914161.1 | E | Italy | rainbow trout |
| 29 | VR/143/Apr14 | MK829701.1 | E | Italy | rainbow trout |
| 30 | TN/509/Oct15 | MK829705.1 | E | Italy | rainbow trout |
| 31 | TN/62/Feb15 | MK829704.1 | E | Italy | rainbow trout |
| 32 | TN/68/Feb15 | MK829703.1 | E | Italy | rainbow trout |
| 33 | TN/86/Feb15 | MK829702.1 | E | Italy | rainbow trout |
| 34 | TN/534/Dec11 | MK829700.1 | E | Italy | rainbow trout |
| 35 | VI/409/Nov06, | MK829696.1 | E | Italy | rainbow trout |
| 36 | TV/310/May05 | MK829693.1 | E | Italy | rainbow trout |
| 37 | TV/3/Dec02 | MK829692.1 | E | Italy | rainbow trout |
| 38 | TN/139/Mar01 | MK829690.1 | E | Italy | rainbow trout |
| 39 | TV/227/2007_L.2 | MK829699.1 | E | Italy | rainbow trout |
| 40 | TV/227/2007_L.1 | MK829698.1 | E | Italy | rainbow trout |
| 41 | TV/310-3.4/May05_L.2 | MK829695.1 | E | Italy | rainbow trout |
